# The HIV-1 Nef Protein Binds Argonaute-2 and Functions as a Viral Suppressor of RNA Interference

**DOI:** 10.1371/journal.pone.0074472

**Published:** 2013-09-04

**Authors:** Madeeha Aqil, Afsar Raza Naqvi, Aalia Shahr Bano, Shahid Jameel

**Affiliations:** International Centre for Genetic Engineering and Biotechnology, Aruna Asaf Ali Marg, New Delhi, India; George Mason University, United States of America

## Abstract

The HIV-1 accessory protein Nef is an important virulence factor. It associates with cellular membranes and modulates the endocytic machinery and signaling pathways. Nef also increases the proliferation of multivesicular bodies (MVBs), which are sites for virus assembly and budding in macrophages. The RNA interference (RNAi) pathway proteins Ago2 and GW182 localize to MVBs, suggesting these to be sites for assembly and turnover of the miRNA-induced silencing complex (miRISC). While RNAi affects HIV replication, it is not clear if the virus encodes a suppressor activity to overcome this innate host response. Here we show that Nef colocalizes with MVBs and binds Ago2 through two highly conserved Glycine-Tryptophan (GW) motifs, mutations in which abolish Nef binding to Ago2 and reduce virus yield and infectivity. Nef also inhibits the slicing activity of Ago2 and disturbs the sorting of GW182 into exosomes resulting in the suppression of miRNA-induced silencing. Thus, besides its other activities, the HIV-1 Nef protein is also proposed to function as a viral suppressor of RNAi (VSR).

## Introduction

The human immunodeficiency virus type 1 (HIV-1) expresses structural (Env, Gag), regulatory (Rev, Tat) and accessory (Nef, Vif, Vpr, Vpu) proteins, of which the last group of proteins are dispensable for virus infection and replication *in vitro*, but are essential for disease progression in the susceptible host [1]. Nef is an ∼27-kDa myristoylated protein that is expressed early during infection and functions as a multifunctional pathogenic factor [2]. It localizes to endosomal and plasma membranes and affects multiple cellular pathways, principally cellular activation, cell survival and apoptosis, and expression of cell surface receptors by virtue of its interaction with multiple cellular proteins [3], [4].

The RNA interference (RNAi) pathway is an innate response that limits viral replication in plants, insects and higher animals [5]–[7]. Unlike in plants where the siRNA pathway is deployed as an antiviral response, animals utilize miRNAs to prevent virus establishment. Many recent reports suggest that the mammalian miRNA pathway functions in antiviral RNA-silencing to restrict the replication of infecting viruses [8]. The knockdown of Dicer, a key miRNA biogenesis protein enhances the replication of influenza A virus [9] and VSV [10]. Interactions between HIV-1 and cellular RNAi pathways not only restrict viral replication but can also promote latency. Human miR-28, miR-29a, miR-125b, miR-150, miR-223, and miR-382 have all been shown to target the 3′ untranslated region (UTR) of HIV-1 transcripts [11]–[13], potentially taking productive infection towards latency. Recent advances in deep sequencing technology have also identified small RNA species derived from the viral genome in HIV-1 infected cells [14]. Further, the knockdown of miRNA biogenesis proteins - Dicer, Drosha or DGCR8 result in increased viral replication [13], [15]. Finally, viral transcripts have been co-localized with RNAi effector proteins in the P bodies [13]. Thus, cellular miRNAs affect HIV-1 replication, either through direct targeting of viral RNAs [16] or through targeting of cellular RNAs necessary for viral replication [17].

Naturally then, these viruses are also expected to encode silencing suppressors to counter this host defense [18]. The HIV-1 Tat protein was reported to suppress RNAi through a direct, RNA-dependent interaction and inhibition of Dicer or, alternatively, through the sequestration of mature miRNAs [17]. It has been suggested that binding of the cellular protein TRBP to the structured TAR elements present in HIV-1 transcripts competitively inhibits the activity of TRBP as a co-factor for Dicer, leading to reduced miRNA processing [19]. However, other evidence supports Tat only as a transcriptional activator, but not as a suppressor of RNAi [20].

The Argonaute protein Ago2, which is an integral component of the miRNA-mediated silencing pathway in animals, localizes predominantly to endosomal membranes [21]. It was recently shown to be present on multivesicular body (MVB) membranes together with GW182, miRNAs and repressed mRNAs [22], [23]. Blocking the maturation of MVBs into lysosomes resulted in enhanced miRNA-mediated silencing, whereas reduced silencing was observed on depletion of the ESCRT proteins, which are required for the maturation of early endosomes to MVBs [24]. Thus, GW-bodies and MVBs appear to be physically and functionally connected with efficient RNAi activity. Together these studies highlight the impact of endosomal pathways on miRNA-mediated silencing [23].

The Nef protein is secreted in the extracellular medium as part of nanovesicles called exosomes [25]. These originate from MVB membranes, contain proteins, mRNAs and miRNAs, and are believed to act as intercellular messengers [26]. Since Nef also localizes to endosomal and plasma membranes and increases the formation of MVBs [27], which are the sites for virus assembly and budding in macrophages [28], we asked whether Nef associates with components of the RISC and perturbs miRNA-mediated silencing. We demonstrate here that Nef directly binds Ago2 through its conserved GW motifs, inhibits its slicing activity and redistributes components of the RISC between cells and exosomes. Thus, Nef acts as a viral suppressor of RNAi (VSR) through a novel mechanism hitherto not observed for any other mammalian VSR protein.

## Materials and Methods

### Plasmids and Cell Lines

Plasmids pMSCV-Puro, pMLV-GagPol, pEYFPN1 and pVSVg were from Clontech. The pEYFPN1-Nef expression vector was previously described [29]. Plasmids pRL-TK-let7a(wt), pRL-TK-let7a(mut) and GW182-EGFP were obtained from Addgene. U937 cells were maintained in RPMI 1640 medium with 10% FBS and antibiotics at 37°C with 5% CO_2_; 350 ng/ml Puromycin was added to this for U937/Nef-EYFP and U937/EYFP stable cell lines. The HEK293T cells were maintained in Dulbecco’s modified Eagle’s medium (DMEM) containing 10% FBS and antibiotics. All cell lines were procured from ATCC.

### Antibodies and Other Reagents

The polyclonal antibody to Nef has been described earlier [29]. The anti-human MHC I hybridoma culture supernatant was from Dr. Satyajit Rath (National Institute of Immunology, New Delhi, India). Anti-Nef MAb-AE6 was from Dr. James Hoxie obtained through the NIH AIDS Research and Reference Reagent Program and human sera 18033 and IC6 for detecting GW and P bodies, respectively, were from Dr. Marvin Fritzler (University of Calgary, Canada). The Ago2 (#32381), GFP (#6556) and GW182 (#84403) antibodies were purchased from Abcam and the Dicer (NBP1-06520) antibody was from Novus Biologicals. The commercial sources for the other antibodies were as follows - anti-CD4-PerCP from BD Biosciences; Biotin anti-CD80 and anti-CD86, and Streptavidin-PE from EBiosciences; antibodies to Actin, Calnexin, VDAC, GAPDH, Alix, Tsg101 and CD81 were from Santa Cruz Biotechnology. For the secondary antibody conjugates, anti-human DyLight 594 was purchased from Abcam, all HRP-conjugated secondary antibodies were from Santa Cruz Biotechnology (USA), and the Alexa dye-conjugated secondary antibodies were from Molecular Probes (USA). Double-stranded siRNA was purchased from Dharmacon (SA). Sephacryl S200HR beads were from GE Healthcare. All PCR primers were purchased from Sigma; common laboratory reagents were from Sigma and Merck.

### Generation of Retroviruses and Stable Cell Lines

Plasmids pEYFP-N1 and pEYFP-Nef-F2 [29] were digested with BamHI and HpaI, and the released fragments containing the *eyfp* and *nef-eyfp* genes respectively were cloned into BglII and HpaI sites in the pMSCV retroviral transfer plasmid. The positive clones were confirmed by restriction digestion and analyzed for EYFP or Nef-EYFP expression by transient transfection in HEK293T cells and western blotting with anti-GFP antibody. Retroviruses expressing Nef-EYFP or EYFP were generated by cotransfection of HEK293T cells in a T25 flask with 2 µg of the transfer plasmid, 1 µg of pGag-Pol and 0.5 µg of pVSVg using the calcium phosphate method. The culture supernatants were collected after 36 hr and used as the source of recombinant retroviruses. Human monocytic U937 cells were washed with RPMI, starved for 90 min without serum and then transduced with 500 µl of culture supernatants per 1×10^6^ cells. After a 4 hr adsorption step, the cells were washed and kept in complete medium for 48 hr prior to the addition of 350 ng/ml puromycin. The cells were split every 48 hr and those surviving after 5 passages were used for the analysis. The clones were sorted for the EYFP positive population using a Becton Dickinson Aria Cell Sorter in the Central Facility of the National Institute of Immunology, New Delhi, India. The sorted clones were cultured for 4–5 passages and checked for purity and YFP expression using Cyan-ADP flow cytometer (Beckman Coulter). Data was analyzed using Summit 4.3 software.

### Characterization of Cell Lines for Surface Markers

Functional characterization of the Nef-EYFP and EYFP stable cell lines was done by assessing the surface expression of various molecules on monocytes that are down regulated by the Nef protein. These include CD4, MHC I, CD80 and CD86; CD54 was used as a negative control. The cells were washed twice with FACS buffer and an appropriate concentration of the primary antibody was added for 45 min on ice. The cells were again washed twice with FACS buffer and stained with 100 µl of 1∶10,000 diluted Streptavidin PE-conjugated secondary antibodies or 1∶5,000 diluted anti-mouse PE-conjugate for 15 min at room temperature. After two washes with PBS the cells were suspended in 500 µl PBS and acquired on a Cyan-ADP flow cytometer (Beckman Coulter). Data was analyzed using the Flow-Jo Software.

### Confocal Microscopy and Colocalization Studies

Multivesicular bodies were labeled in U937 cells by exogenous delivery of the fluorescently labeled lipid marker N-rhodamine-labeled phosphatidylethanolamine (NRhPE) [30]. Briefly, U937 cells stably expressing Nef-EYFP or EYFP were cultured in complete RPMI at 37°C and 5% CO_2_ for 30 min in the presence of 5 µM NRhPE. Cells were harvested and washed twice with PBS at 2000 rpm and 4°C for 5 min each. Live cells were mounted using antifade containing DAPI (Invitrogen, Carlsbad, CA, USA). To study the colocalization of Nef with Ago2, the U937 cells stably expressing Nef-EYFP were permeabilized using the FACS permeabilizing buffer for 20 min on ice. Cells were pelleted at 2000 rpm for 5 min at 4°C. Primary antibodies (rabbit anti-Ago2, mouse anti-Nef or human IC6 sera) were added at a 1∶100 dilution in the same buffer and incubated on ice for 45 min. Cells were then washed twice with permeabilizing buffer and stained with Alexa-conjugated secondary antibody diluted 1∶500 in the same buffer. After washing, the cells were fixed in 0.5% paraformaldehyde and mounted using antifade containing DAPI (Invitrogen, Carlsbad, CA, USA). Images were acquired using a Nikon A1/R confocal microscope at 60× magnification. To determine colocalization of Nef-EYFP with MVBs, Ago2 and P Bodies, the images were quantified using the JACoP plugin in Image J software.

### Immunoprecipitation Studies

About 6 million cells were lysed in 600 µl of lysis buffer (Cell Signaling Technology). Lysates were normalized for protein content and 500 µg of total proteins in 500 µl of lysis buffer were incubated with 25 µl of Protein A-agarose beads for 1 hr at 4°C. The pre-cleared lysate was then incubated with 2 µg of the antibody overnight at 4°C, followed by 40 µl of Protein A-agarose beads for 2 hr at 4°C. After five washes with lysis buffer, the beads were boiled in 2× SDS-PAGE sample buffer and western blotting was performed.

### Construction Nef GW Mutants

Mutagenesis of the Nef GW motifs was carried out using the Quick-Change site-directed mutagenesis kit (Stratagene) following the manufacturer’s protocol. Two single mutants W13A and W141A and a double mutant W13,141A were generated using the pMT3-Nef plasmid as a template, and the following primers: W13A-F, GCATAGTTGGAGCGCCTGATATAAGA; W13A-R, CTTATATCAGGCGCTCCAACTATG CTG; W141A-F, CACTGACTTTTGGGGC GTGCTTCAAGC; and W141A-R, GCTTGAAGCACGCCCCAAAAGTCAG. All mutants were subsequently verified by sequencing.

### Gel Filtration Assay

Sephacryl S200HR beads (GE Healthcare) were packed in a 3 ml column. Total lysates were prepared from ∼10 million cells in 50 µl Buffer D that contained 20 mM HEPES, pH 7.9, 0.2 mM EDTA, 0.5 mM DTT, 50 mM KCl, 10% glycerol, 0.2 mM PMSF and 0.5× Protease inhibitor cocktail (Roche). The cells were lysed with 5 cycles of sonication on ice, each with 25% power for 5 sec followed by cooling for 55 sec. The lysates were clarified by centrifugation at 10,000×g for 10 min at 4°C. The protein content was estimated by the Bradford assay and 150 µg protein in a volume of 50 µl was passed through the column pre-equilibrated with PBS. Fractions of 3 drops (∼120 µl) each were collected, precipitated with acetone and separated by SDS-PAGE. Western blotting was done with anti-Ago2 and anti-EYFP antibodies.

### HIV-1 p24 Assay

The p24 levels in transfected culture supernatants were quantified using the HIV-1 p24^CA^ Antigen Capture Assay Kit (NCI-Frederick Cancer Research and Development Center), according to the supplier’s instructions.

### HIV-1 Infectivity Assay

About 0.5 million 1G5-Jurkat indicator cells were infected with 100 ng p24 equivalents of various viruses in 1 ml of RPMI lacking serum. After a 4 hr incubation at 37°C in a 5% CO_2_ incubator, the cells were washed twice with this medium. The cells were then incubated at 37°C in a 5% CO_2_ incubator in 12-well plates in 1 ml of RPMI-10% FBS. The cells were harvested after 48 hr, lysates were prepared and luciferase activity was measured using the Dual Luciferase Assay System (Promega Corporation, Madison, USA), following the manufacturer’s protocol. The luciferase activity was measured using a luminometer (Sirius, Berthold, Germany).

### Functional Assay for miRNA Activity

To study the functional effects of Nef on the miRNA pathway, we performed co-transfection experiments using wild type and GW mutants of Nef, with a plasmid expressing the *Renilla* mRNA with a 3′UTR containing two hsa-miR-let7a target sites (pRLTK-let7a(wt)), or a negative control containing two seed mismatches (pRLTK-let7a(mut)). Co-transfection of the luciferase reporter plasmids and expression vectors for either wild type or GW mutant Nef was carried out in 293T and U937 cells using Fugene 6 and nucleofection, respectively, and plasmid pGL3 (containing firefly luciferase) as a transfection control. Briefly, 100 ng of pRLTK-let7a(wt) or let7a(mut) luciferase reporter DNA, 700 ng of Nef or control DNA and 100 ng of pGL3 DNA were transfected in 293T cells in 12-well plates. Nucleofection of exponentially growing U937 cells was carried out using Amaxa nucleofector solution as per supplier’s instructions, and the cells plated into wells of 6-well plates in 3 ml RPMI-10% FBS. The cells were harvested 48 hr post-transfection and luciferase assay was performed using the Dual Luciferase Assay Kit (Promega, Madison, USA), according to the supplier’s protocol.

### RISC Loading Assay

Double stranded siRNA oligonucleotides (Dharmacon) were 5′ end-labeled with ^32^P-γ-ATP (Perkin Elmer) and polynucleotide kinase (Fermentas) and purified using G-25 columns (GE Healthcare). For the siRNA loading experiment, 1.25 pmole of siRNA was incubated in a 35 µl reaction with 25 µg of the cell lysate in binding buffer containing 50 mM Tris-HCl pH 8.8, 50 mM glycine, 8% glycerol and 2 mM DTT for 30 min at 4°C. The lysate was prepared in Buffer D as described above. For competition, a 50-fold molar excess of unlabeled siRNA duplex was included in the reaction. The samples were mixed with loading dye and resolved on a 6% non-denaturing polyacrylamide gel in 1× TBE for 2 hr at 200V and 4°C. The gel was dried and imaged using a Typhoon scanner. For the immunodepletion assay, 400 µg of pre-cleared lysates were first incubated with 5 µg of either anti-Ago2 or anti-Dicer antibody at 4°C overnight, followed by precipitation with 50 µl Protein A/G beads. These immunodepleted lysates were then used in the binding assay described above.

### Slicer Assay

The slicer assay was performed with immunopurified Ago2 and an *in vitro* transcribed, ^32^P-labelled 124 nt let7a target RNA as described elsewhere [31] with some modifications. To immunopurify Ago2, 100 million U937/Nef-EYFP or U937/EYFP cells were lysed with 3×5 sec pulses of sonication at 30% amplitude in a buffer containing 30 mM HEPES, pH 7.4, 150 mM KOAc, 2 mM Mg(OAc)_2_, 5 mM dithiothreitol (DTT), 0.1% Nonidet P-40 and EDTA-free Protease Inhibitor Cocktail (Roche). The lysate was pre-cleared with Protein G Sepharose beads before incubation with an anti-Ago2 antibody (Abcam) for overnight at 4°C. Thereafter, 50 µl of Protein G Sepharose was added and mixed for 2 hr at 4°C. The beads were washed twice with lysis buffer containing 5M NaCl followed by a wash with 1× cleavage buffer, which contained 25 mM Hepes-KOH, pH 7.5, 50 mM KOAc, 5 mM Mg(OAc)_2_, 5 mM DTT, 10 mM creatine phosphate and 0.5 mM ATP. For *in vitro* synthesis of the target RNA, the 3′UTR of let7a was first cloned as a SacII-SpeI fragment in plasmid pGEMTEasy. The plasmid was linearized with NdeI and *in vitro* transcription was carried out at 37°C for 2 hr in a 20 µl reaction containing 0.5 µg linear DNA, 0.5 mM all rNTPs except UTP, which was added at a concentration of 15 µM, 5 µl α^32^P UTP (10 mCi/ml) and 2 µl T7 RNA polymerase (Epicenter). The 124 nt RNA was purified from a 8% polyacrylamide gel containing 7M urea. A 20 µl cleavage reaction was set up at 37°C for 90 min with 5,000 cpm of the ^32^P-labelled let7a RNA and Ago2 immunoprecipitates in 1× cleavage buffer containing 40 units RNAsin, 0.5 ug yeast RNA and 10 units of Creatine phosphokinase (Sigma). The cleavage products were separated on an 8% polyacrylamide gel containing 7 M urea, and the gel exposed on a phosphorimager screen in the Typhoon scanner. A 5′ end-labeled 59 nt oligonucleotide was run on the gel as a size marker. The gel image was quantified with the NIH Image J software and densitometric analysis was carried out to determine the percentage of cleaved products.

### Preparation and Characterization of Exosomes

Exosomes were prepared from cells that were cultured in media depleted of exosomes present in fetal calf serum. For preparing exosome-depleted media, RPMI containing 50% FCS was subjected to ultracentrifugation at 100,000×g and 4°C for 16 hr, followed by filtration through a 0.22 µm filter. This was stored at 4°C and diluted appropriately with RPMI prior to use. The U937/Nef-EYFP and U937/EYFP cells were grown in RPMI-10% FCS. Culture supernatants were collected after two days and exosomes were prepared following the basic protocol-1 described elsewhere [32]. All steps were carried out at 4°C. Briefly the culture supernatant was first centrifuged at 300×*g* for 10 min to pellet cells. Dead cells were removed by centrifugation at 2,000×*g* for 10 min followed by removal of cell debris by centrifugation for 30 min at 10,000×g. The clarified supernatant was centrifuged at 100,000×g in a SW28 rotor for 1 hr. The exosome pellet was washed by suspension in 30 ml of PBS and pelleting as above. The final pellet was suspended in 100 to 200 µl of PBS and frozen at −70°C.

The protein content of exosomes was estimated using the Bradford assay. The appropriate amount of exosomes was mixed with 6× SDS loading dye, boiled for 5 min and the proteins separated by SDS-PAGE. Western blotting was then carried out using antibodies against standard exosomal marker proteins - Alix, Tsg101 and CD81, at 1∶1000 dilutions. Antibodies against various other proteins were used to rule out any contamination from subcellular organelles and membranes - Calnexin (ER), VDAC (mitochondria) and Cytochrome C (apoptotic bodies), at 1∶4000 dilutions. Rabbit anti-Nef and anti-GFP were used at 1∶3000 and 1∶4000 dilutions, respectively.

For electron microscopy, carbon-coated Formvar grids of 300 µm mesh size were floated on a drop of 5 µg/ml exosome sample for 30 sec. Excess solution was wicked away from the grids with Whatman paper and these were then placed for 60 sec on a drop of 1% uranyl acetate that had been filtered through a 0.45 µm filter. Excess stain was wicked away and grids were imaged on a FEI, Tecnai, T-12 (G2 Spirit) Transmission Electron Microscope operating at 120 kv.

## Results

### Nef Colocalizes with MVBs and Argonaute 2 in U937 Cells

**Figure 1:**
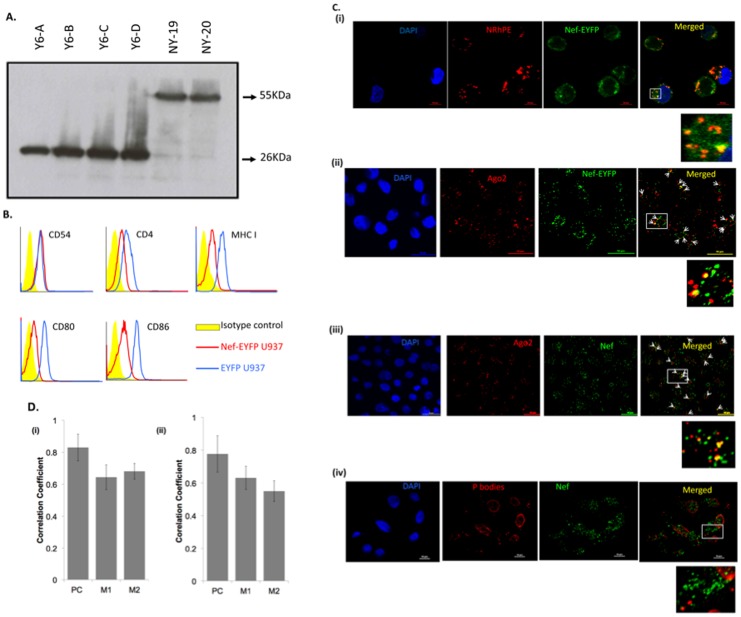
Characterization of Nef-expressing cell lines. U937 cells stably expressing either a Nef-EYFP fusion protein or EYFP were established as described in Materials and Methods. (A) Western blot for expression of EYFP (lanes Y6-A to -D) and Nef-EYFP (lanes NY-19 and -20) in selected U937 stable clones. (B) Flow cytometric analysis of U937/Nef-EYFP and U937/EYFP cells for surface expression of the indicated proteins. (C) The U937/Nef-EYFP cells were (i) cultured with 5 µM NRhPE, or (ii) stained with anti-Ago2 as described in Materials and Methods. U1 cells were activated with PMA and labeled with anti-Nef and either (iii) anti-Ago2 or (iv) anti-P bodies as described in Materials and Methods. The white arrowheads in the merged image represent colocalization points. The images are representative of three independent experiments. The boxed regions are expanded. (D) Correlation analysis of the colocalization of Nef with Ago2 in (i) U937/Nef-EYFP cells and (ii) activated U1 cells showing Pearson’s coefficient (PC) and Manders coefficients (M1, M2). The coefficients represent an average of three independent images, each consisting of at least 10 cells, calculated using the JACoP plugin within the Image J software. M1 represents the fraction of Ago2-red overlapping with Nef-green and M2 represents the fraction of Nef-green overlapping with Ago2- red. Bars represent ± SD.

We generated U937 human monocytic cells that stably expressed either a Nef-EYFP fusion protein or EYFP. Western blotting with anti-GFP antibodies showed several independent cell pools to express proteins of the expected size (Fig. 1A). The surface levels of CD4, MHC I, CD80 and CD86 estimated by flow cytometry were reduced on U937/Nef-EYFP cells compared to U937/EYFP cells (Fig. 1B). As a control, surface CD54 levels showed no reduction on U937/Nef-EYFP cells. This confirmed that the Nef-EYFP fusion protein expressed in U937 cells was functional. Since Nef localizes to intracellular membranes [27], we examined its colocalization with MVBs and Ago2. Nef-EYFP (Fig. 1C–i) but not EYFP (not shown) colocalized with NRhPE in the U937 stable cell lines and also in HEK293T cells that ectopically expressed Nef-EYFP (Fig. S1A). Endogenous Ago2 also showed significant colocalization with Nef-EYFP in the cell lines (Fig. 1C–ii) as well as in activated U1 human monocytic cells [33] that produce infectious HIV and express Nef from the proviral genome (Fig. 1C–iii). However, Nef did not colocalize with P bodies that are also sites for miRNA-mediated silencing [34] (Fig. 1C–iv). Another important component of miRISC is GW182, which is present in association with Ago2 in GW bodies on MVB membranes [13], [24]. Since Ago2 interacts directly with GW182 [34], we checked whether Nef and GW182 also colocalize. In HEK293T cells co-transfected with plasmids expressing the Nef-DsRed and GW182-EGFP fusion proteins, no colocalization was observed (not shown). These results suggest that Nef is closely associated with some components of GW bodies on MVB membranes. We carried out quantitative analysis by determining the Pearson correlation and Manders correlation for colocalization of Nef with Ago2 in the U937/Nef-EYFP cells (Fig.1D–i) and activated U1 cells (Fig.1D–ii). The Pearson coefficient (PC) of 0.83 in U937/Nef-EYFP cells and 0.77 in activated U1 cells indicates colocalization of Nef and Ago2 in both cell lines. The Manders coefficient M1 represents the fraction of Ago2-red overlapping with Nef-green and M2 represents the fraction of Nef-green overlapping with the Ago2- red. These values also indicate similar levels of Ago-2 colocalizing with Nef and vice-versa in cells that either express Nef-EYFP alone or express Nef from the viral genome together with HIV-1 proteins.

### Nef Interacts with Ago2 but not GW182

**Figure 2:**
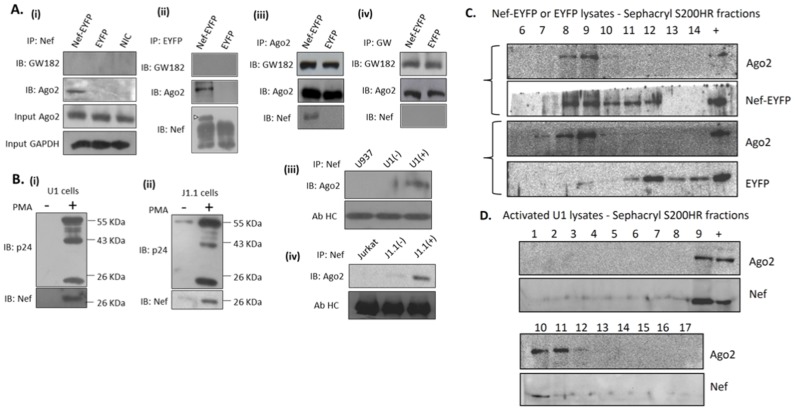
Nef interacts with Ago2. (A) Lysates of U937/Nef-EYFP or U937/EYFP cells were subjected to immunoprecipitation (IP) followed by immunoblotting (IB) with the indicated antibodies. The lane marked NIC represents IP of U937/Nef-EYFP lysates with an irrelevant antibody. (B) U1 and J1.1 cells were treated with DMSO (–) or PMA (+) and cell lysates were either immunoblotted for p24 and Nef (i, ii), or IP/IB with anti-Nef and anti-Ago2 (iii, iv), as described in Materials and Methods. U937 and Jurkat cells served as background controls and antibody heavy chain as loading control. (C) Lysates were prepared from the U937 stable cell lines using Buffer D as described in Materials and Methods. From this, 150 µg lysate was passed through a pre-equilibrated Sephacryl S200HR column with a 3 ml bed volume. The column was eluted and fractions of 3 drops (∼120 µl) were collected, which were then precipitated with acetone, separated by SDS-PAGE and western blotting was done with Ago2 and GFP antibodies. (D) Lysates from activated U1 cells were prepared and fractionated as in (C) followed by SDS-PAGE and western blotting with Ago2 and Nef antibodies. The profiles shown are representative of three separate experiments.

To test whether Nef and Ago2 interact, we performed co-immunoprecipitations with Nef, Ago2 and EYFP antibodies. Anti-Nef antibodies precipitated Ago2 but not GW182 from Nef-EYFP cells; no such precipitation was observed from control EYFP cells (Fig. 2A–i). To rule out an EYFP-Ago2 interaction, we performed immunoprecipitation with anti-EYFP antibodies and obtained similar results as with anti-Nef antibodies (Fig. 2A–ii). When immunoprecipitations were carried out with anti-Ago2 antibodies, Nef was detected in the precipitates from Nef-EYFP cells but not from control cells (Fig. 2A–iii). Anti-Ago2 antibodies also precipitated GW182 from Nef-EYFP as well as control cells (Fig. 2A–iii). Finally, anti-GW182 precipitated Ago2 from both cell lines, but did not precipitate Nef (Fig. 2A–iv). These results show that Nef interacts with Ago2 but not with GW182 or the Ago2-GW182 complex. We then validated the Nef-Ago2 interaction in HIV-1 infected cells. Latently infected U1 (monocytic) and J1.1 (CD4+ T) cells when activated with phorbol-3-myristic acid (PMA) expressed high levels of p55Gag, its processing intermediates, the p24 capsid and Nef proteins (Fig. 2B–i,ii). Immunoprecipitates of Nef from activated U1 and J1.1 cells also contained Ago2 (Fig. 2B–iii,iv). Finally, lysates prepared from U937/Nef-EYFP, U937/EYFP or activated U1 cells were fractionated on a Sephacryl S200HR gel permeation column and the eluted fractions were western blotted to detect Ago2 and Nef. A significant fraction of Nef-EYFP co-eluted with Ago2 at the column void volume, while this was not the case with EYFP (Fig. 2C). Similarly, Nef expressed from the virus background in U1 cells also co-eluted with Ago2 at the column void volume (Fig. 2D). Together these experiments show that Nef interacts with Ago2, either when stably expressed in U937 cells, or when expressed from integrated HIV-1 genomes in cells of monocytic and CD4+ T lineages.

### Conserved GW Motifs in Nef are Required for Ago2 Binding and Efficient Viral Replication

**Figure 3:**
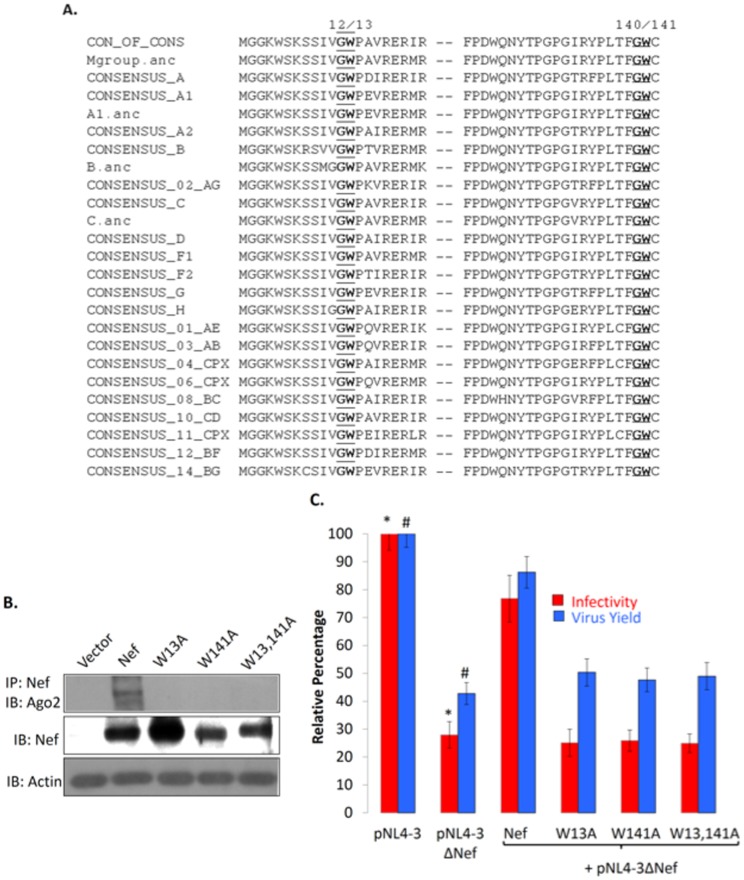
Mutations in Nef GW motifs abrogate its interaction with Ago2 and reduce virus yield and infectivity. (A) Alignment of Nef sequences. All 2660 Nef amino acid sequences available in the Los Alamos HIV database were analyzed. Regions encompassing the two conserved GW motifs (bold and underline) at positions 12–13 and 140–141 are shown for consensus and ancestral (.anc) sequences for various HIV-1 M group clades (A–H), and consensus sequences for various recombinant circulating forms. (B) HEK293T cells were transiently transfected to express wild type or GW mutant Nef proteins. Cell lysates were precipitated with anti-Nef and blotted with anti-Ago2. Nef and Actin were expression and loading controls, respectively. (C) HEK293T cells were transfected with either pNL4-3 or pNL4-3ΔNef; cells were also co-transfected with pNL4-3ΔNef and expression vectors for either the wild type or GW mutant Nef proteins. After 36 hr the culture supernatants were quantified for p24. Purified viruses equivalent to 100ng p24 were also used to infect 0.5 million 1G5 Jurkat indicator cells, and 48 hr later the cells were harvested, lysed and luciferase assay was performed as described in Methods. Error bars represent mean ± SD from three independent experiments, and p-values calculated using the Student’s *t*-test were as follows: * 0.002; # 0.023.

Proteins such as mammalian GW182 that interact with Argonaute have multiple GW/WG motifs [35]. Some plant and insect viruses that encode VSR proteins, which bind Argonaute, require at least two GW motifs for the interaction [36], [37]. Two Trp-binding pockets were also characterized in the recently elucidated structure of mammalian Ago2 [38]. The HIV-1 NL4-3 Nef sequence contains two GW motifs at residues 12–13 and 140–141, respectively. To test for conservation, we compared 2660 Nef amino acid sequences belonging to HIV-1 of different clades and recombinant circulating forms within Group M as well as HIV-1 Groups N and O, available in the Los Alamos HIV Sequence Database. The N-terminal and C-terminal GW motifs showed 92% and 95% conservation, respectively, but were completely conserved in consensus and ancestral sequences for various HIV-1 subtypes and recombinant circulating forms (Fig. 3A).

To investigate the importance of GW motifs in the Nef-Ago2 interaction, we generated single and double GW→GA mutants in the Nef protein by site-directed mutagenesis. Expression plasmids for wild type Nef, the two single mutants (W13A; W141A) and the double mutant (W13,141A) were transiently transfected into HEK293T cells. While all the proteins expressed at comparable levels, only the wild type Nef but neither the single nor double GW mutants pulled down Ago2 from transfected cell lysates (Fig. 3B). This confirmed that both the GW motifs in Nef are important for its interaction with Ago2, and a mutation in either abrogates this binding.

We then explored if the Nef-Ago2 interaction has functional effects on HIV-1 replication and infectivity. For this, we transfected HEK293T cells with pNL4-3 or pNL4-3ΔNef; the latter was also cotransfected with expression vectors for wild type or GW mutant Nef, and virus yields were estimated by quantifying p24 in the transfected cell supernatants. As reported earlier [39], the *nef*-deleted infectious clone showed ∼50% reduction in virus yield, which was *trans*-complemented by wild type but not GW-mutant Nef proteins (Fig. 3C). To assess for the infectivity of these viruses we infected the 1G5 Jurkat indicator cell line with equal p24 amounts of virus and carried out luciferase reporter assays after 2 days as described in Methods. Compared to the wild type virus, the *nef*-deleted virus showed only about 30% infectivity. However, the infectivity was almost completely restored when the *nef*-deleted virus was trans-complemented with wild type Nef but not with any of the mutant Nef constructs (Fig. 3C). Thus, Nef mutants that do not interact with Ago2 are also neither fully replication competent nor fully infectious, suggesting that the Nef-Ago2 interaction is important for HIV-1 replication and infectivity.

### Nef Suppresses miRNA-induced Silencing

**Figure 4:**
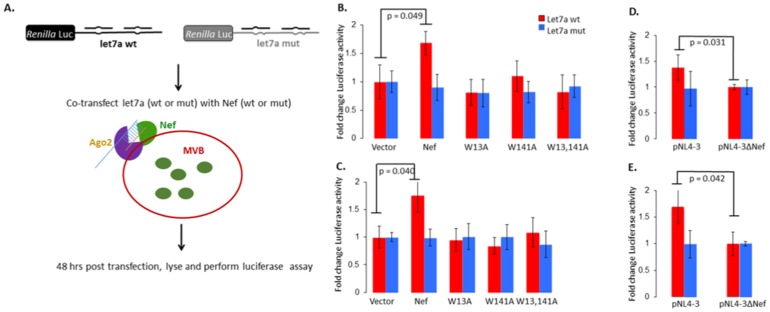
Nef suppresses miRNA-mediated silencing. (A) Schematic representation of the luciferase based miRNA functional assay using plasmid pRLTK-let7a containing the *Renilla* luciferase gene with wild type or mutant miRNA let7a binding sites in the 3′UTR, and expression plasmids for wild type or GW mutant Nef proteins. The transfections were carried out in (B) U937 and (C) HEK293T cells. Transfections of pRLTK-let7a and either pNL4-3 or pNL4-3ΔNef were also carried out in (D) U937 or (E) HEK293T cells. After 48 hr the cell lysates were prepared and assayed for luciferase activity. Constitutively expressed firefly luciferase readings were used for normalization. Error bars represent mean ± SD from three independent experiments. P-values were calculated using Student’s *t*-test.

To study the effects of Nef on miRNA-mediated silencing, we carried out a *Renilla* luciferase (RLuc)-let-7a reporter assay as outlined in Figure 4A [15]. The let-7a miRNA was chosen because it is enriched at MVB membranes [22]. U937 (Fig. 4B) or HEK293T (Fig. 4C) cells co-transfected with the Nef expression vector and the RLuc-let7a reporter showed ∼70% increase in luciferase activity compared to the empty vector, which implies a reduction in let7a activity in the presence of Nef. Such an increase was observed neither with the mutant let7a reporter nor with the Nef GW mutants. To test this silencing suppression activity of Nef in the context of HIV-1 replication, we co-transfected the RLuc-let7a reporters with either pNL4-3 or pNL4-3ΔNef in U937 or HEK293T cells. There was ∼40% suppression in U937 cells (Fig. 4D) or ∼60% suppression in HEK293T cells (Fig. 4E) of let7a miRNA activity by wild type HIV-1 compared to *nef*-deleted HIV-1. There was no effect of Nef or its mutants on ectopically expressed luciferase mRNA (not shown). Together these results show that Nef suppresses miRNA-mediated silencing and its GW motifs are required for this activity. We conclude that through its interaction with Ago2, Nef alters miRISC activity at the MVB surface.

### Nef Inhibits the Slicing Activity of Ago2

**Figure 5:**
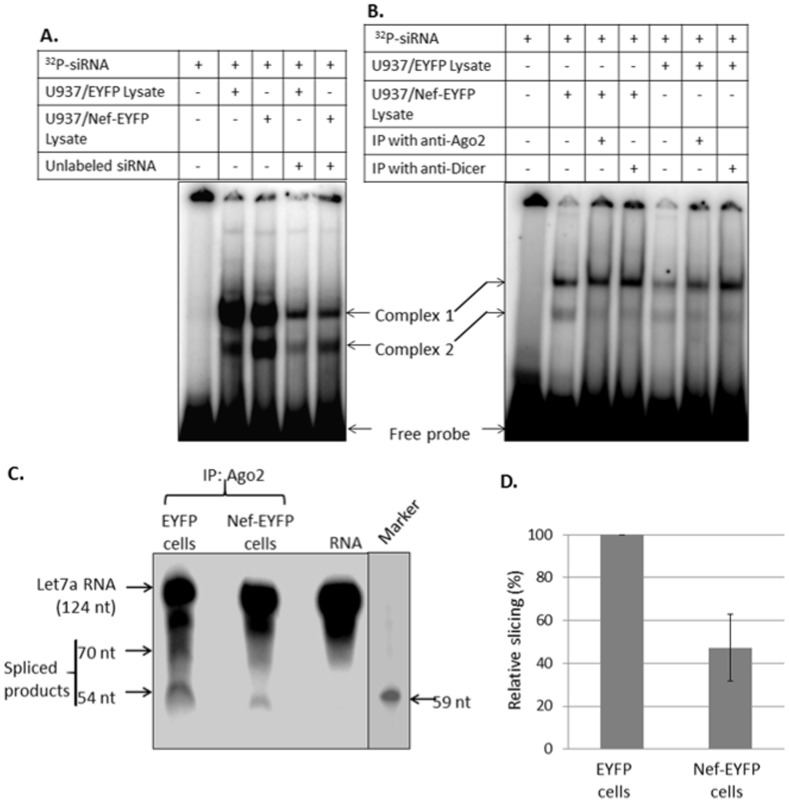
Nef does not affect siRNA loading into RISC, but inhibits its slicing activity. (A) Lysates from the U937 stable cell lines were prepared in Buffer D as described in Materials and Methods. From this, 25 µg of lysate was incubated with ^32^P-labeled duplex siRNA (without or with 50-fold molar excess of unlabeled siRNA) in binding buffer for 30 min at 4°C. The complexes were separated on a 6% non-denaturing polyacrylamide gel. (B) The gel shift assay was set up as in (A) except that lysates immunodepleted for either Ago2 or Dicer were also included. The positions of the mobility shifted complexes and free probe are indicated. (C) Slicer activity was assayed using immunoprecipitated Ago2 from U937/Nef-EYFP or U937/EYFP cell lysates and a ^32^P-labeled *in vitro* transcribed let7a RNA, as described in Materials and Methods. The positions of full-length RNA and the two sliced products are shown. The 59 nt marker oligonucleotide was run on the same gel, but a lower exposure is shown. (D) Densitometric analysis of autoradiograms from three independent experiments was carried out using Image J (version 1.4.1). The slicing activity in Nef-EYFP lysates is represented as a percentage of that in EYFP lysates.

To understand the mechanism of suppression, we checked whether Nef affects loading of small RNAs in the RISC and/or mRNA slicing activity of Ago2. Extracts from the U937/Nef-EYFP and U937/EYFP cells were incubated with ^32^P-labeled duplex siRNA and the complexes were analyzed on a 6% polyacrylamide gel. Two complexes were observed as reported earlier [40], but neither showed reduction in the presence of Nef (Fig. 5A). While both complexes were competed out with an excess of unlabeled duplex siRNA (Fig. 5A), only Complex 2 was reduced following immunodepletion of Ago2 or Dicer (Fig. 5B), suggesting that this represented the RISC. Thus, Nef does not affect the loading of Ago2 with small RNAs. This observation also supports the hypothesis that loading of Ago2 with miRNA occurs on membranes other than MVBs [23]. The effect of Nef on the slicing activity of Ago2 was then tested with Ago2 immunoprecipitates from U937/Nef-EYFP and U937/EYFP cell lysates, using a radiolabeled 124 nt let-7a mRNA. Besides the intact let-7a RNA, three other RNA species were observed, which included the two expected cleavage products of 70 nt and 54 nt (Fig. 5C). The cleavage was quantified by dividing the combined intensities of the 70 nt and 54 nt bands by the total intensities of all four bands in the two lanes. This analysis showed less than 50% cleavage of let-7a RNA by Ago2 immunoprecipitates from U937/Nef-EYFP cells compared to U937/EYFP cells (Fig. 5D). Thus, while Nef does not appear to affect the loading of miRNA in the miRISC, it reduces the ability of Ago2 to slice the target mRNA.

### Nef Perturbs the Sorting of GW182 into Exosomes

**Figure 6:**
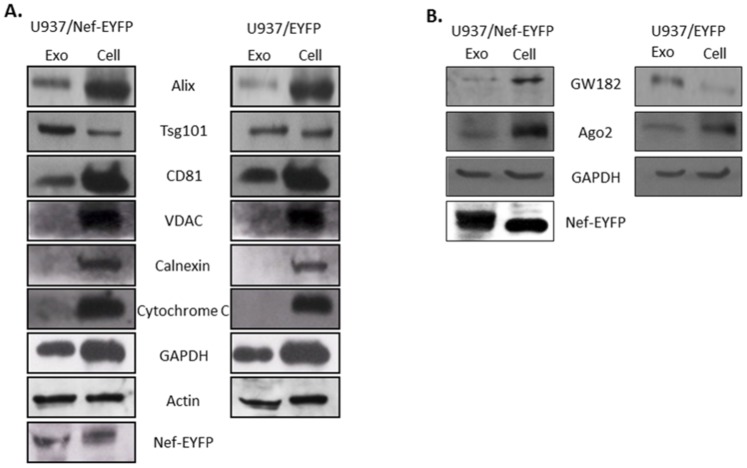
Nef blocks the sorting of GW182 into exosomes. Exosomes were purified from the spent culture media of the two cell lines as detailed in Materials and Methods. (A) Western blotting was done using 40 µg of cellular and exosomal lysates using antibodies that detect marker proteins for exosomes (Alix, Tsg101, CD81), mitochondria (VDAC), endoplasmic reticulum (Calnexin) and apoptotic bodies (Cytochrome C). GAPDH and Actin were used as loading controls and anti-GFP antibodies were used to detect Nef-EYFP and EYFP. (B) Cellular and exosomal lysates were subjected to SDS-PAGE and western blotting to detect GW182, Ago2 and Nef; GAPDH was used as a loading control.

The physical association of GW bodies and MVBs allows for the sorting of GW182 into exosomes; blocking this secretion results in accumulation of inactive RISC and reduced miRNA activity [22]–[24]. The absence of GW182 in Nef immunoprecipitates led us to investigate the distribution of Ago2 and GW182 in Nef-expressing cells and exosomes released from these cells. Exosomes were isolated from the culture supernatants of the two cell lines and characterized for identity and purity by western blotting. The exosome preparation was positive for the proteins Alix, Tsg101 and CD81, which are exosomal markers, but was negative for markers of mitochondria (VDAC), endoplasmic reticulum (Calnexin) and apoptotic bodies (Cytochrome C) (Fig. 6A). Cellular GAPDH, Actin and Nef, previously shown to be present in exosomes, were also detected in our preparation. The exosomes were visualized by negative staining transmission electron microscopy as 50–100 nm membrane limited vesicles (data not shown). Besides the U937 stable cell line, Nef was also present in exosomes prepared from HEK293T cells that transiently expressed this protein (Fig. S1B).

While there was no significant size difference between exosomes secreted by U937/Nef-EYFP and U937/EYFP cells, the former secreted about 50–75% more exosomes than the latter (data not shown). This was observed earlier for T cells as well [25], [41]. The expression of Ago2 and GW182 and their distribution into exosomes were then compared for Nef-expressing and control cells. There were higher levels of Ago2 and GW182 in U937/Nef-EYFP cells compared to U937/EYFP cells, but similar amounts of the Ago2 proteins were detected in the exosomes from these cells (Fig. 6B). However, compared to exosomes from U937/EYFP cells that are rich in GW182, exosomes from U937/Nef-EYFP cells had very low levels of GW182, even though high amounts are expressed in these cells (Fig. 6C). This aberrant distribution of GW182 is also likely to affect miRISC turnover and activity.

## Discussion

In this study we show that HIV-1 Nef functions as a viral suppressor of RNA interference (VSR). This is attributed to its localization to MVBs and its interaction with Ago2, a critical component of the miRISC. Nef increases the proliferation of MVBs in various cell types [27], and these are also the sites for HIV assembly in monocytes and macrophages [28]. The virus buds into the MVB lumen and is stored protected from immune surveillance, thus making monocytes and macrophages important viral reservoirs. A significant fraction of the Nef-EYFP protein colocalizes with NRhPE, a lipid marker for MVBs. We also found Nef to colocalize with Alix, a component of the ESCRT complex (data not shown), which is present on the MVB surface and is important for vesicular trafficking in late endosomes. Interestingly, Nef was shown earlier to interact with AIP, another ESCRT complex protein, and to promote virus release [39]. Recently, MVBs were also suggested to be sites of miRNA-mediated silencing. It was shown that GW bodies containing Ago2 and GW182 physically interact with MVBs and are independent RNAi sites in addition to P bodies [22], [24]. Our results show that Nef and Ago2 interact and are part of a larger complex.

The GW182 protein interacts with Ago2 through its multiple GW motifs that are also called AGO hooks [37]. Turnip Crinckle Virus p38 is a newly identified VSR that has two GW-motifs at its N- and C-termini [36]. Similarly, we found two highly conserved GW motifs in Nef from HIV-1 types M, N and O and SIVcpz. Close to the N-terminal GW motif, a cluster of highly conserved arginine residues in Nef at amino acid positions 17, 19, 21 and 22 mediate its RNA binding activity and its secretion in exosomes [42], [43]. The interaction of Nef with Ago2 was lost when either one or both of its GW motifs were mutated. These mutations also affected the replication and infectivity of HIV produced from an infectious molecular clone. Being one of the earliest viral proteins to be expressed, we propose that Nef attenuates host RNA interference and thus promotes HIV replication.

Other VSR proteins inhibit RNAi either by interfering with RISC loading through small RNA sequestration or by binding and inhibiting the slicing activity of Argonaute. For example, the influenza virus NS1 and vaccinia virus E3L proteins sequester small interfering RNAs, thus inhibiting the loading of RISC [44]. The cricket paralysis virus 1A protein inhibits the Ago2 slicing activity associated with the RISC [45]. We did not find any significant effect of Nef on the loading of a small double-stranded in the RISC, but found it to inhibit the slicing activity of the RISC. The partial effect of Nef on the overall slicing activity within the RISC (∼50% inhibition) can be explained by its subcellular localization, thus affecting only the silencing associated with GW bodies present on MVBs. However, in the context of HIV infection, this might significantly suppress the silencing effects of hitherto unidentified cellular antiviral miRNAs that function preferentially within GW bodies to restrict HIV replication. A recent study on the role of RNAi proteins in HIV infection showed that viral replication is not affected by the knockdown of some P body proteins except Ago2 [39]. This suggests that even when P bodies are disrupted, the AGO proteins in GW bodies can modulate HIV infection.

The secretion of GW182 into exosomes is important for the turnover of RISC and effective miRNA-mediated silencing in mammalian and insect cells [22], [24]. Nef increases exocytosis and is itself secreted into exosomes [25], [39]. We now show that exosomes from Nef-expressing cells carry reduced levels of GW182, a majority of which is retained in the cells; however, Nef did not significantly affect the partitioning of Ago2 between cells and exosomes. Thus, Nef competes with GW182 for binding to Ago2 and affects the sorting and release of GW182 into exosomes. The Nef-Ago2/miRNA complex is akin to an inactive RISC. This was established through a functional miRNA silencing suppression assay, in which only Nef proteins that could bind Ago2 suppressed the silencing of a luciferase mRNA carrying let-7a binding sites in its 3′UTR.

In conclusion, our results identify Nef as a viral suppressor of miRNA-mediated silencing. This can be attributed to its MVB localization and binding to Ago2 through conserved GW motifs, previously observed only in VSR proteins from plant and insect viruses. We propose that this interaction destabilizes the miRNA-mRNA-Ago2 complex, inhibits the slicing activity of Ago2 and hinders the sorting of GW182 into exosomes. This would be a novel mechanism for RNAi suppression, hitherto not observed in any mammalian virus. Since exosomes also package mRNAs and miRNAs [46], it would be interesting to see whether Nef regulates the exosomal sequestration and export of specific RNAs towards comprehensive effects on HIV-infected cells and on intercellular communication. We are currently exploring these effects of Nef towards its role in HIV pathogenesis.

## Supporting Information

Figure S1 **Nef colocalizes with MVBs and is secreted in exosomes from HEK293T cells.** (A) HEK293T cells were transiently transfected with the Nef-EYFP expression vector and 36 hr post-transfection cells were cultured in the presence of 5 µM NRhPE for 30 min. Imaging was performed on live cells without fixation. (B) HEK293T cells were transfected with the Nef expression plasmid pMT3-Nef or the control plasmid pMT3. After 48 hr, culture supernatants were harvested and exosomes were isolated using the differential centrifugation protocol. Total exosomal protein (40 ug) was separated by SDS-PAGE followed by western blotting to detect either CD81 (exosomal marker) or Nef.
(TIFF)
