# Supplementary figures and images for: The HIV-1 Nef Protein Binds Argonaute-2 and Functions as a Viral Suppressor of RNA Interference
Source: PLoS One. 2013 Sep 4;8(9):e74472. doi: 10.1371/journal.pone.0074472 (PMC3762824; doi:10.1371/journal.pone.0074472)

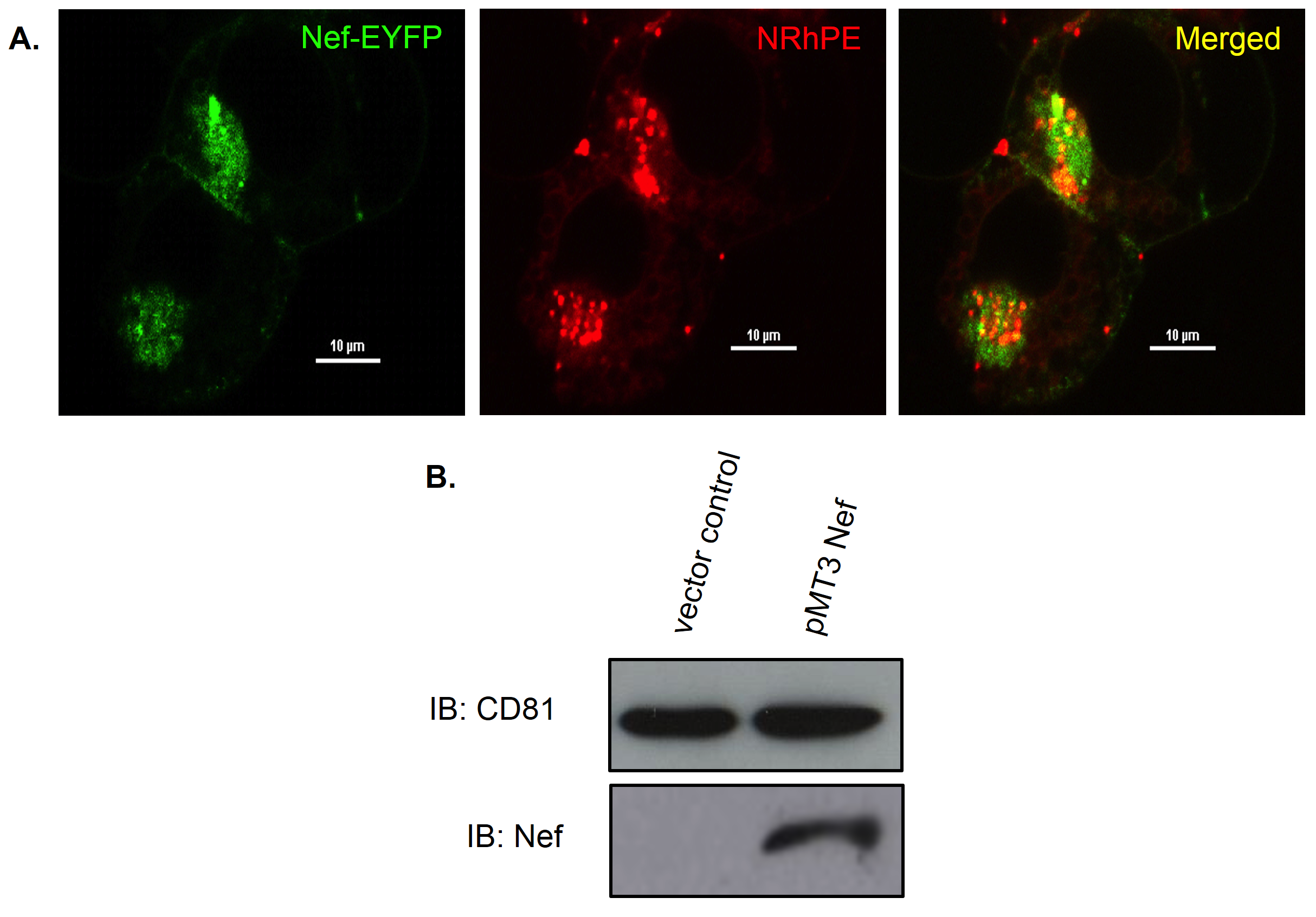

Supplement: Figure S1 — Nef colocalizes with MVBs and is secreted in exosomes from HEK293T cells. (A) HEK293T cells were transiently transfected with the Nef-EYFP expression vector and 36 hr post-transfection cells were cultured in the presence of 5 µM NRhPE for 30 min. Imaging was performed on live cells without fixation. (B) HEK293T cells were transfected with the Nef expression plasmid pMT3-Nef or the control plasmid pMT3. After 48 hr, culture supernatants were harvested and exosomes were isolated using the differential centrifugation protocol. Total exosomal protein (40 ug) was separated by SDS-PAGE followed by western blotting to detect either CD81 (exosomal marker) or Nef. (TIFF) [file pone.0074472.s001.tiff]
